# Influence of Double-Pulse Electrodeposition Parameters on the Performance of Ag/AgCl Electrochemical Electrode for Marine Electric Field

**DOI:** 10.3390/s24072103

**Published:** 2024-03-25

**Authors:** Chenjuan Wang, Yuguo Li, Yunju Wu

**Affiliations:** 1 College of Marine Geosciences, Ocean University of China, Qingdao 266100, China; chenjuanwang@126.com; 2 Key Laboratory of Submarine Geosciences and Prospecting Techniques of Ministry of Education, Ocean University of China, Qingdao 266100, China; wuyunju@stu.ouc.edu.cn

**Keywords:** double-pulse electrodeposition method, positive direction peak current density, deposition time, Ag/AgCl marine electric field electrode

## Abstract

This paper describes a Ag/AgCl electrochemical electrode for marine electric field measurements, which was prepared by depositing silver chloride on a silver foil substrate using double-pulse electrodeposition. The impact of positive direction peak current density and deposition time on electrode performance in the preparation of Ag/AgCl electrodes through double-pulse electrodeposition was investigated. Scanning electron microscopy and voltammetry have been used to study the properties of the prepared electrodes. This work reveals the correlation between the electrochemical behavior of electrodes and the physical properties of their surfaces, especially specific surface area and porosity. The study verified the characteristics of Ag/AgCl marine electric field electrodes obtained with different pulse parameters by analyzing the potential stability and noise level of the electrode in salt water. The study’s results have positive significance for improving the accuracy of marine electric field measurements.

## 1. Introduction

The electric field in natural seawater contains a wealth of information [1]. Currently, marine electric field detection technology is widely used in environmental monitoring, marine resource exploration, etc. [2,3]. Electrochemical electrodes are the core devices for marine electric field measurement [4,5,6,7,8,9,10]. The electric field in seawater is a stable current field, which is also a potential field. Electrodes are used in pairs for marine electric field measurements [4]. In the field, the marine electric field instrument measures the electric field between two electrodes that are usually 10 m apart and are attached at the ends of an arm. The electric field strength between the two electrodes is determined by the potential difference recorded by the pair of electrodes [10]. The equation for calculating the electric field strength (*E*) is as follows:

(1) E=∆U/∆d

where ∆*U* is the potential difference recorded by the electrode pair, and ∆*d* is separation between the paired electrodes, typically 10 m.

The electric field signal in seawater is mainly at low to medium frequencies and is very weak due to attenuation [11,12]. To obtain more electric field information, it is necessary to use low-noise electrodes to conduct long-term measurements in seawater, and the tests usually last several days. This also means that the electrode must have long-term potential stability. Low noise means that the electrochemical noise generated by the electrode is very little when the electrode potential, exchange current density, and other states fluctuate randomly and out of equilibrium with time. If the noise of the electrode is too high, the marine electric field signal will be overwhelmed and no useful marine electric field signal can be obtained [13]. Long-term potential stability refers to when the potential fluctuation of each electrode of the pair is essentially consistent during the test process, thereby reducing the effect of the electrode pair’s potential difference drift on the test findings [14]. A solid non-polarized Ag/AgCl electrode is one of the most widely used electrodes for marine electric field measurements [4,13,15]. This electrode can quickly establish electrochemical equilibrium with chloride ions in seawater that enable the electrode to have high exchange current density, good electrochemical reversibility and potential stability, as well as low noise level in seawater [16,17]. Compared with other types of electrodes such as $Cu/CuSO_{4}$ and carbon fiber electrode, a Ag/AgCl electrode is more suitable for marine electric field measurement [18,19].

Commonly used preparation methods for Ag/AgCl marine electric field electrodes include direct current electrolysis, thermal dip plating, and sintering [20,21,22,23]. Among them, the direct current electrolysis method allows rapid preparation of Ag/AgCl marine electric field electrodes by applying constant current (i.e., chronopotentiometry) or potential (i.e., chronoamperometry) to cover the substrate surface with a AgCl deposition layer. It has been shown that the morphology of electrodes, such as specific surface area and porosity, plays an important role in electrode performance [24]. The specific surface area is the factor that links particle-scale characteristics to macro-scale physical and chemical properties [25,26,27], and its magnitude is inversely proportional to particle size [28]. Larger porosity allows the electrode to have a higher reaction surface area. The electrode’s large specific surface area and porosity allow for a higher exchange current density and response rate under equilibrium conditions, as well as improved electrode potential stability. However, too much porosity will also increase solution penetration, bringing the electrolyte into contact with the electrode substrate, thereby increasing the possibility of generating a mixed electromotive force. Moreover, the surface of the electrode substrate serves as a high-free-energy surface, and impurities may also be adsorbed to the interface during the preparation of the electrode, which may also have a significantly variable impact on the potential of the electrode. Therefore, in order to achieve potential stability of the Ag/AgCl electrode, it is necessary to control the porosity of the prepared electrode.

It is reported that compared with the direct current electrolysis method, the double-pulse electrolysis method can refine the particle size and increase the density of the deposited layer [29,30]. The double-pulse electrodeposition method uses pulse current or voltage generated by a pulse power supply to improve the surface condition of the deposited layer, which can not only quickly and efficiently prepare electrodes with stable performance, but also overcome the problems of an electrode prepared by direct current electrolysis. However, this method has not been widely used for the preparation of Ag/AgCl marine electric field electrodes. Therefore, in this paper, the double-pulse electrodeposition method is used to prepare electrodes and study their properties.

Parameters such as the positive direction peak current density and deposition time of the double-pulse electrodeposition are believed to affect the stability of the Ag/AgCl electrode. In this paper, we control the morphology of the electrode by adjusting the parameters in the electrode preparation process, and then control the performance of the electrode. From experimental results, we first demonstrated the impact of two important parameters, positive direction peak current density and deposition time, on the electrode morphology, and then investigated the relationship between the morphology of the deposition layer and the properties of the electrode. Based on these experiments, the double-pulse electrodeposition parameters for preparing electrodes with good stability and noise level were obtained. This work aims to explore the influence of double-pulse electrodeposition parameters on electrode performance, so as to develop the best method for the preparation of Ag/AgCl marine electric field electrodes with excellent performance.

## 2. Experiments

### 2.1. Pretreatment of Silver Foil Substrate

**Figure 1:**
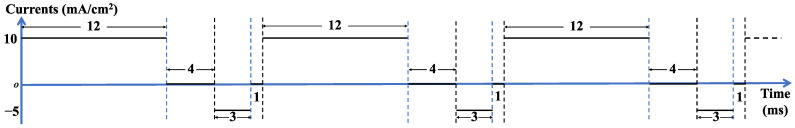
Variations in peak current density and duty cycle with time for the electrodeposition process.

Silver foil (99.998%, Thermo Fisher Scientific, Sunnyvale, CA, USA) was used as the substrate for preparing electrodes, with dimensions of 5 cm × 10 cm × 0.1 mm. The substrate was pretreated at room temperature and protected from light. The pretreatment included silicon carbide paper polishing, reagent soaking and cleaning, and double-pulse electrodeposition. Firstly, the silver foil was sanded with 1500-grade silicon carbide paper. Secondly, the silver foil was immersed in acetone for 6 h and then ultrasonically cleaned in acetone, ethanol, and deionized water for 30 min. Finally, a double-pulse power supply (i.e., high-frequency pulse rectifier, SDD12V5000mA, Kingsunny, Shenzhen, China) was used for electrodeposition. Figure 1 shows the waveform of the double-pulse electrodeposition and the parameters of the double-pulse electrodeposition; the positive direction peak current density ($i_{an}$) and the reverse direction peak current density ($i_{p}$) were 10 $mA/{cm}^{2}$ and 5 $mA/{cm}^{2}$, the positive pulse length ($t_{on}$) and the pulse pause ($t_{off}$) were 12 ms and 4 ms, the reverse pulse length and the duty cycle (i.e., $\theta =t_{on}/(t_{on}+t_{off})$) were 3 ms and 0.75, and the deposition time was 250 s.

### 2.2. Preparation of Ag/AgCl Electrochemical Electrode by Double-Pulse Electrodeposition

The silver foil was welded to the wires of the underwater connector at room temperature and protected from light, then encapsulated in an electrode protective shell and the solder joint was sealed with epoxy resin. The electrode protection shell is 15.9 cm in height and 6.8 cm in diameter, and is made of a polyformaldehyde (POM) shell and a polypropylene (PP) cotton lining. POM has excellent corrosion and friction resistance, while PP cotton has excellent light shielding, corrosion resistance, and water permeability. The space between the electrode protective shell and the silver foil was filled by the mixed powder of AgCl and diatomite with a volume ratio of 1:6, in which AgCl powder (99.997%, Thermo Fisher Scientific, Sunnyvale, CA, USA) was used as the active material and diatomite (AR, Thermo Fisher Scientific, Sunnyvale, CA, USA) as filler. Once the mixed powder filling was completed, the electrode protection shell was covered and packaged. After immersion in 3.5% sodium chloride solution, the encapsulated electrode was subjected to double-pulse electrodeposition. The double-pulse electrodeposition parameters for preparation of the electrode are shown in Table 1.

**Figure 2:**
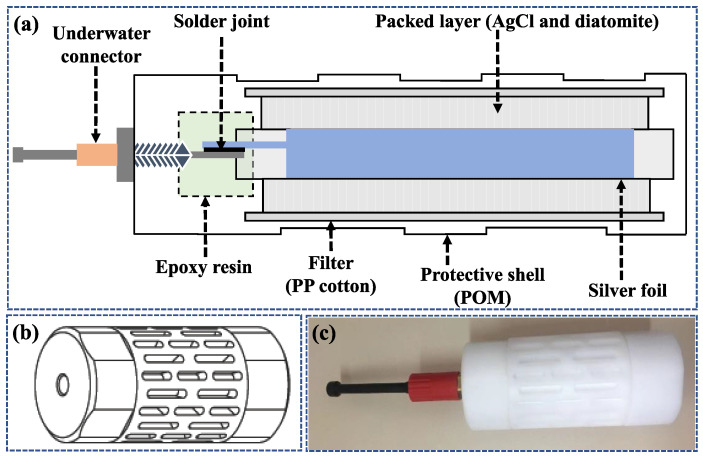
(**a**) Schematic diagram of the internal structure of the electrode; (**b**) structure diagram of the protective shell; (**c**) picture of the electrode.

The schematic diagram of the internal structure of the electrode, the structure diagram of the protective shell, and the picture of the electrode are shown in Figure 2a, 2b, and 2c, respectively. As we can see in Figure 2b, there are a series of long holes in the protective shell, which are channels for material exchange between the electrode and the electrolyte (salt water). The filtration precision of the PP cotton in the electrode is 5 μm. Therefore, the substances inside and outside the electrode can be exchanged through the holes of the protective shell and the PP cotton.

### 2.3. Characterization

The surface morphology of the Ag/AgCl electrode’s deposition layer was investigated with a Zeiss Gemini 300 (Carl Zeiss, Jena, Germany) Scanning electron microscope (SEM).

At room temperature, the exchange current density of the electrode was tested using a ParStat 4000A electrochemical workstation (AMETEK Scientific Instruments, Berwyn, PA, USA). During the test, the working electrode (WE) was the prepared Ag/AgCl electrochemical electrode, the reference electrode (RE) was a saturated calomel electrode (SCE), and the counter electrode (CE) was a Pt sheet. The electrode exchange current density was measured in potentiodynamic mode, with the scan range of −0.5 V to 0.5 V and the scan rate of 0.5 mV/s.

## 3. Results and Discussion

### 3.1. Morphological Characterization of the Electrode

**Figure 3:**
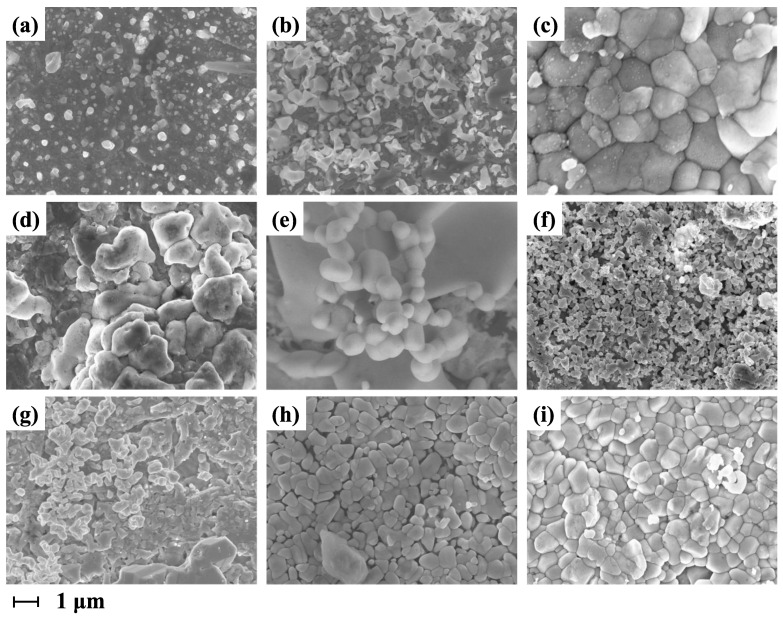
SEM images of Ag/AgCl electrode plated at a deposition time of 300 s: (**a**) $i_{an}=5\;mA/{cm}^{2}$, (**b**) $i_{an}=7.5\;mA/{cm}^{2}$, (**c**) $i_{an}=10\;mA/{cm}^{2}$, (**d**) $i_{an}=12.5\;mA/{cm}^{2}$, (**e**) $i_{an}=15\;mA/{cm}^{2}$; SEM images of Ag/AgCl electrode plated at a positive direction peak current density of $10\;mA/{cm}^{2}$: (**f**) t=220 s, (**g**) t=240 s, (**h**) t=260 s, (**i**) t=280 s.

The photomicrographs of the Ag/AgCl electrode deposited layer at five different values of positive direction peak current density with the same deposition time and five different values of deposition time with the same positive direction peak current density are shown in Figure 3. The SEM examination of the Ag/AgCl electrode deposited at different positive direction peak current density and deposition time revealed the fact that the morphology of the deposited layer is strongly influenced by the double-pulse electrodeposition parameters. It is obvious that the distribution and morphology of the particles (i.e., shape and size) in the deposited layer are different due to different electrodeposition parameters. The characteristics of the particles in the electrode’s deposition layers prepared with different double-pulse parameters are summarized in Table 2.

According to the analysis and discussion of electrocrystallization by Budevski et al. [31], the grain size and growth pattern are determined by the interaction of nucleation rate and growth rate. In the double-pulse electrodeposition experiments, the morphology of the deposited layer is determined by deposition time and positive direction peak current density. Hence, the size and number of particles in the deposited layers obtained under different electrodeposition parameters vary.

As shown in Figure 3a–e, the density and particle morphology of the deposited layer changed significantly with the increase in the positive direction peak current when the deposition time was kept at 300 s. The deposited layer obtained at lower positive direction peak current density (Figure 3a) has smaller-sized particles and the silver chloride formed does not yet completely cover the available surface area. This phenomenon increases the permeability of the solution, thereby increasing the possibility of generating a mixed electromotive force. In addition, the content of AgCl, which is the main raw material for marine electric field electrodes, affects the lifetime of the electrode. The AgCl content in the electrode deposition layer prepared under these conditions (Figure 3a) is limited, which will make the lifetime of the electrode very limited. Under higher positive direction peak current density (Figure 3e), the uniformity of particle size in the deposited layer was significantly reduced, containing a higher proportion of larger-sized particles and exhibiting a coarser grain structure. Therefore, the difference in performance between individual electrodes prepared under this condition can be relatively large.

The deposited layers of electrodes prepared with a positive direction peak current density of 10 $mA/{cm}^{2}$ and different deposition time are shown in Figure 3f (t = 220 s), Figure 3g (t = 240 s), Figure 3h (t = 260 s), Figure 3i (t = 280 s), and Figure 3c (t = 300 s). The size and porosity of the deposited layer particles changed with deposition time, but the change in porosity is more pronounced. The electrode prepared with the shortest deposition time (Figure 3f) is characterized by a porous but non-uniform particle size of the deposition layer with different AgCl contents in each area. When the deposition time was 240 s (Figure 3g) and 260 s (Figure 3h), although the electrode deposition layer had a certain porosity, the size of the particles in the deposition layer varied greatly. The uneven distribution of particle sizes in the deposited layer may lead to poor electrochemical performance of the electrode. For the electrode prepared with a deposition time of 280 s (Figure 3i), the multi-layered deposition layer has a uniform particle size and some porosity. As the deposition time was further increased to 300 s (Figure 3c), the particle size uniformity of the deposited layer decreased, and the specific surface area and porosity decreased. This leads to a reduction in the reaction area, which may also reduce the reaction rate of the electrode.

### 3.2. The Exchange Current Density of the Electrode

Exchange current density measurements were performed to verify previous conclusions and to investigate the effects of deposition time and positive direction peak current density on the performance of the prepared Ag/AgCl electrode.

The interface between the electrode and the electrolyte solution is the location of the chemical reaction. The exchange current density ($i_{0}$) at the interface is a measure of the electrode’s electrocatalytic activity, which can control the physical processes that occur at the interface and the electrode, as well as reflects the degree of electrode polarization and the difficulty of the electrode process [32]. Electrodes with higher exchange current density are less prone to polarization and have better stability and response rates. The morphology of the particles in the electrode deposition layer controls the exchange current density of the electrode [33], while the reaction area of the deposited layer and the concentration of the reactants also plays a role. The reaction area of Ag/AgCl electrode prepared by double-pulse electrodeposition is concentrated on the electrode’s surface, and its size is proportional to the specific surface area of the particles and the number of particles per unit area. The specific surface area is an important characteristic of the electrode and is inversely proportional to particle size [28]. When the particle size of the deposited layer is smaller, the specific surface area increases. As a result, its surface activity is higher, and surface phenomena such as chemical reaction rate and adsorption become more significant. In addition, the electrode reaction area is proportional to the number of particles per unit area for particles of the same size.

**Figure 4:**
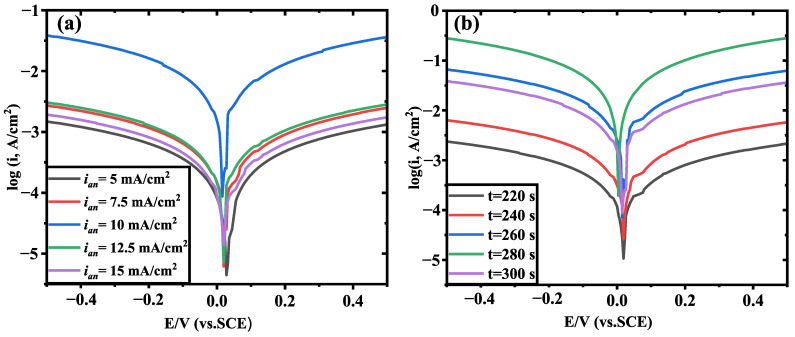
(**a**) Polarization curves of Ag/AgCl electrode plated at five different values of positive direction peak current density with the same deposition time (t=300 s); (**b**) polarization curves of Ag/AgCl electrode plated at five different values of deposition time with the same positive direction peak current density ($i_{an}=10\;mA/{cm}^{2}$).

Figure 4a shows the polarization curves of the Ag/AgCl electrode plated at the same deposition time (t = 300 s) with five different values of positive direction peak current density, $i_{an}=5\;mA/{cm}^{2}$ (black line), $i_{an}=7.5\;mA/{cm}^{2}$ (red line), $i_{an}=10\;mA/{cm}^{2}$ (blue line), $i_{an}=12.5\;mA/{cm}^{2}$ (green line), and $i_{an}=15\;mA/{cm}^{2}$ (purple line). Figure 3b displays the polarization curves of the Ag/AgCl electrode plated at the same positive direction peak current density ($i_{an}=10\;mA/{cm}^{2}$) with five different values of deposition time, t = 220 s (black line), t = 240 s (red line), t = 260 s (blue line), t = 280 s (green line), and t = 300 s (purple line, same as the blue line of Figure 4a). The exchange current density of the electrode varies depending on the electrodeposition parameters. As shown in Figure 4a, the exchange current density of the electrode is larger, when prepared with a positive direction peak current density of $i_{an}=10\;mA/{cm}^{2}$ and a deposition time of 300 s (Figure 4a blue line). Compared with Figure 4b, it can be seen that when the positive direction peak current density of the prepared electrodes is the same ($10\;mA/{cm}^{2}$) and the deposition time is 280 s, the exchange current density of the obtained electrode is larger than others (Figure 4b green line). However, when the positive direction peak current density is $10\;mA/{cm}^{2}$, the electrode with a deposition time of 280 s (Figure 4b green line) has a greater exchange current density than the electrode with a deposition time of 300 s (Figure 4b purple line, i.e., Figure 4a blue line). The results indicate that the Ag/AgCl electrodes prepared with the positive direction peak current density of $10\;mA/{cm}^{2}$ and the deposition time of 280 s exhibit the maximum exchange current density.

From the morphology test results of electrodes prepared with different parameters (Figure 3), it can be seen that the deposited layer of the electrode has a good morphology when the peak current density is 10 $mA/{cm}^{2}$ and the deposition time is 280 s. Furthermore, the electrodes obtained under these conditions exhibit better exchange current density. Therefore, the test results of electrode exchange current density are consistent with the morphology of the deposited layer.

### 3.3. The Potential Stability of the Electrode

During the electrode process, ions are transferred from the solution phase to the electrode phase, resulting in the generation of an interphase potential between the two phases. The electrode potential is made up of the interphase potential and the electrode surface potential [34]. The property of an electrode is determined by its structure. Changes in electrode potential can provide useful evidence to explain morphology differences in electrodes. According to the Nernst equation, the potential of the Ag/AgCl electrode prepared in this paper can be expressed as

(2) $$U_{AgCl/Ag}=\varphi_{{Ag}^{+}/Ag}^{\Theta }+\frac{RT}{F}ln\frac{K_{sp(AgCl)}^{\Theta }}{C_{{Cl}^{-}}}$$

where $\varphi_{{Ag}^{+}/Ag}^{\Theta }$ represents the standard potential of the Ag/AgCl electrode, *F* is the Faraday constant, R is the molar gas constant, *T* is the thermodynamic temperature, $K_{sp(AgCl)}^{\Theta }$ is the solubility product constant, and $C_{{Cl}^{-}}$ is the concentration of ${Cl}^{-}$ in the electrolyte. For electrode 1 and electrode 2, which have the same raw materials and preparation processes, their potentials are also the same under standard conditions (i.e., $U_{AgCl/Ag,1}=U_{AgCl/Ag,2}$). However, due to slight differences in the process of preparing the electrodes, there are certain differences in the structures of electrode 1 and electrode 2, and their potentials are not exactly the same (i.e., $U_{AgCl/Ag,1}\neq U_{AgCl/Ag,2}$). This results in a potential difference between electrode 1 and electrode 2. In general, the smaller the potential difference between the electrode pairs, the better the long-term potential stability.

From Equation (1), it can be seen that the potential difference is proportional to the electrode separation. To reduce the effect of environmental electromagnetic interference on electrode potential stability, a minimum distance should be maintained between electrode pairs during electrode potential stability testing experiments. As shown in Equation (2), temperature and chloride ion concentration have an effect on the potential of the Ag/AgCl electrochemical electrode. In order to test the effects of temperature and chloride ion concentration on the potential stability of the electrode, we conducted the following experiments.

At room temperature, the electrodes were immersed in 3.5% NaCl solution. Equation (2) can be simplified as follows:

(3) $$U_{AgCl/Ag}=0.7991-1.89\times 10^{-3}T\;(V)$$

**Figure 5:**
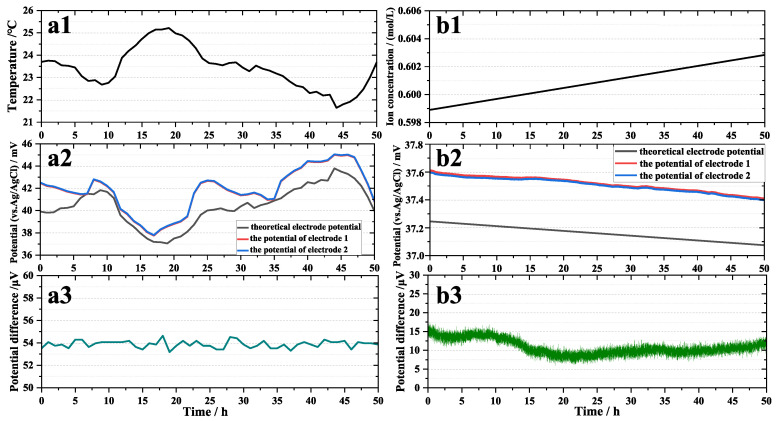
Temperature (**a1**) and chloride ion concentration (**b1**) curve, temperature-dependent (**a2**) and chloride ion concentration-dependent (**b2**) fluctuation curve of self-made electrode potential and theoretical electrode potential, temperature-dependent (**a3**) and chloride ion concentration-dependent (**b3**) fluctuation curve of electrode pair potential difference.

**Figure 6:**
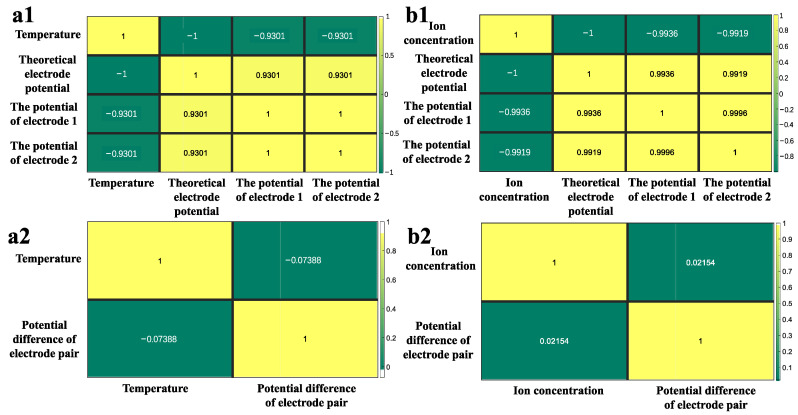
Pearson correlation coefficient between test conditions (temperature (**a1**) and chloride ion concentration (**b1**)) and theoretical electrode potential, potential of electrode 1, and potential of electrode 2, Pearson correlation coefficient between test conditions (temperature (**a2**) and chloride ion concentration (**b2**)), and potential difference of electrode pair.

Figure 5(a1) is the temperature fluctuation curve with time during the test. Figure 5(a2) shows the electrode potential; the black line is the theoretical electrode potential, the red line is the potential of electrode 1, and the blue line is the potential of electrode 2. The theoretical electrode potential that varies with temperature could be calculated using Equation (3). Figure 5(a3) shows the potential difference between electrode 1 and electrode 2, also called the potential difference of the electrode pair. The changes in the potential of the two electrodes with temperature were fairly consistent, as shown in Figure 5(a2), but there was a slight deviation from the theoretical electrode potential. The potential difference of the pair of electrodes was considerably distinct from the temperature change trend. The Pearson correlation coefficient (PCC) [35] was used to demonstrate the temperature dependency of the electrode potential and the potential difference of the electrode pair. From Figure 6(a1), we can see that the PCC between temperature and the theoretical electrode potential, the potential of electrode 1, and the potential of electrode 2 were −1, −0.9301, and −0.9301, respectively. Thus, temperature has a strong negative association with electrode potential. However, the PCC between temperature and the electrode pair’s potential difference (Figure 6(a2)) was −0.07388, and there was basically no correlation between them.

Similarly, at a constant temperature of 25 °C, Equation (2) can be simplified to

(4) $$U_{AgCl/Ag}=0.2218-0.0592logC_{{Cl}^{-}}(V)$$

The Ag/AgCl electrode potential is thus determined by the chloride ion concentration. The theoretical electrode potential (the black line in Figure 5(b2)) could be computed from the chloride ion concentration during the test (Figure 5(b1)). The potentials of electrodes 1 (the red line in Figure 5(b2)) and 2 (the blue line in Figure 5(b2)) were tested simultaneously when measuring the chloride ion concentration. Although the electrode potential and the theoretical potential are slightly different, their change trends were basically the same. The variation in the potential difference of the electrode pair appears to be independent of the chloride ion concentration. The PCC was also used to illustrate the dependence of the electrode potential and the potential difference of the electrode pair on chloride ion concentration. The PCC between the chloride ion concentration and the theoretical electrode potential, the potential of electrode 1, and the potential of electrode 2 were 1, 0.9936, and 0.9919 (Figure 6(b1)). However, the PCC between the chloride ion concentration and the potential difference of the electrode pair (Figure 6(b2)) was 0.02154. It is clear that the chloride ion concentration affects the potential of a single electrode but has little effect on the potential difference of a pair of electrodes.

In summary, the potential of the electrode changes with temperature and chloride ion concentration, but the fluctuation of the electrode pair’s potential difference has little relationship with them. This also proves the consistency of the performance of the electrode prepared by the double-pulse electrodeposition method.

**Figure 7:**
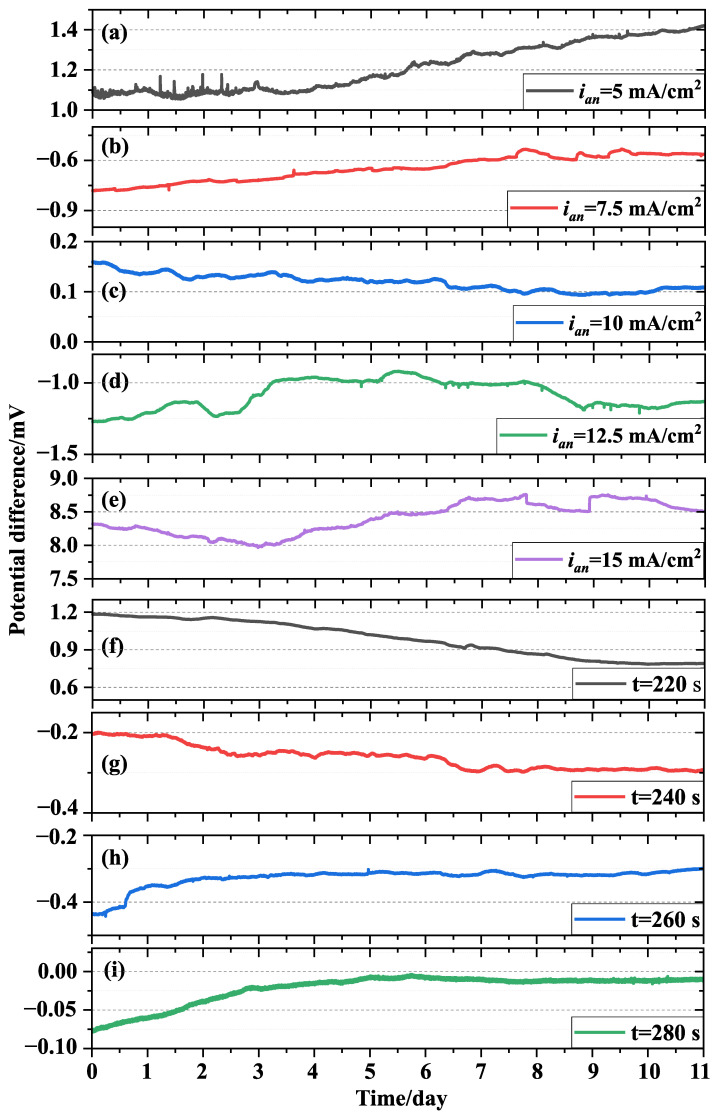
Time series of the potential difference of Ag/AgCl electrode plated at a deposition time of 300 s: (**a**) $i_{an}=5\;mA/{cm}^{2}$, (**b**) $i_{an}=7.5\;mA/{cm}^{2}$, (**c**) $i_{an}=10\;mA/{cm}^{2}$, (**d**) $i_{an}=12.5\;mA/{cm}^{2}$, (**e**) $i_{an}=15\;mA/{cm}^{2}$; time series of the potential difference of Ag/AgCl electrode plated at a positive direction peak current density of $10\;mA/{cm}^{2}$: (**f**) t=220 s, (**g**) t=240 s, (**h**) t=260 s, (**i**) t=280s.

This paper investigated the potential stability of electrodes prepared with different double-pulse parameters by evaluating the potential difference of electrode pairs prepared with the same formula in 3.5% NaCl solution. In order to test the potential stability (including potential difference and potential difference drift) of the electrode prepared with different double-pulse electrodeposition parameters, the potential differences of electrodes prepared by different positive direction peak current density and deposition time were tested for 11 days under the same experimental conditions. For the test, all electrodes were placed in a single water tank containing an appropriate amount of 3.5% NaCl solution. The potential difference of the electrode pair was collected by an Agilent 34972A data acquisition device. The test results are presented in Figure 7. The electrode pairs obtained with different double-pulse electrodeposition parameters have different potential difference and potential difference drift. This confirms the sensitivity of the Ag/AgCl electrode prepared using the double-pulse electrodeposition method to the positive direction peak current density and deposition time employed. Table 3 displays the potential stability parameters of the Ag/AgCl electrode obtained using various double-pulse electrodeposition parameters. In this analysis, we consider both the maximum and minimum values of the electrode pair potential difference and potential difference drift, not just the average value of the potential difference. All tests were conducted under the same experimental conditions to be able to compare the potential stability of electrode pairs prepared with difference double-pulse parameters.

Figure 7a–e display the time series of potential difference for the electrode pairs prepared with different positive direction peak current densities at a deposition time of 300 s. Table 3 shows that the Ag/AgCl electrode prepared with a deposition time of 300 s and a positive direction peak current density of 10 $mA/{cm}^{2}$ exhibits a small potential difference and potential difference drift. This indicates that the electrode pair prepared under this condition has better potential stability. However, the potential difference and potential difference drift of electrode pairs obtained at other parameters are larger. When the positive direction peak current density is 15 $mA/{cm}^{2}$, the potential difference of the electrode pairs is the largest. This indicates that the potential of each electrode prepared under this condition has a large difference. As shown in Figure 3e, the particle size of the electrode deposition layer prepared under this condition varies greatly, and it is difficult to ensure the consistent morphology for all electrodes prepared. This can result in poor reproducibility of performance of electrodes prepared under these conditions. Therefore, there are significant differences in the potential of the electrode prepared at this condition.

Additionally, apart from the positive direction peak current density of 10 $mA/{cm}^{2}$, the potential difference time series of the electrode pairs prepared with different positive direction peak current densities exhibits significant bulges. This is particularly evident in Figure 7a for days 0–3. Combined with the morphology test results of the electrode (Figure 3a), it can be seen that the surface of the electrode substrate prepared under this condition was not completely covered by AgCl particles. When the electrolyte and the electrode are in complete contact, the electrode exhibits a mixed electromotive force, leading to a significant alteration in the potential difference between the electrode pairs.

Figure 7c,f–i show that the potential stability of the electrode pairs obtained at different deposition times is different. As a result, deposition time is an important factor affecting electrode potential stability. Table 3 shows that the Ag/AgCl electrode prepared with the deposition time of 280 s and the positive direction peak current density of 10 $mA/{cm}^{2}$ has the smallest potential difference and potential difference drift. This indicates that the electrode has better potential stability compared with other electrodes, and the electrode prepared with this deposition parameter has better performance repeatability. The potential stability of the electrode prepared with different deposition times can be explained by the morphology. The test results indicate that the electrode prepared at a deposition time of 280 s and a positive direction peak current density of 10 $mA/{cm}^{2}$ has a higher exchange current density and a better morphology of the deposited layer. The potential stability is consistent with the test results for the electrode morphology and exchange current density. Therefore, the morphology of the electrode deposition layer is a crucial factor affecting the electrode potential. For electrodes of the same size and prepared by the double-pulse electrodeposition method, as the morphology of the deposited layer becomes more consistent, the potential difference decreases, resulting in a more consistent performance of the electrode pair. Under identical environmental conditions, electrodes with the same performance maintain consistent potentials, and the potential difference between the electrode pairs remains essentially constant. Otherwise, the potential difference of the electrode pair will fluctuate greatly.

In summary, the morphology of the particles in the electrode deposition layer is an important factor that affects the potential stability of the electrode pair. Larger porosity of the sedimentary layer particles can increase the reaction area of the electrode, but excessive porosity will lead to abnormal fluctuations in the electrode potential. The reaction area of the electrode increases as the particle size of the deposited layer decreases. When the particle size distribution of the deposited layer is more consistent, the potential difference of the electrode becomes smaller. Therefore, the electrode pair’s potential difference over time (i.e., potential difference drift) visually represents its potential stability and response rate under the same experimental conditions.

**Figure 8:**
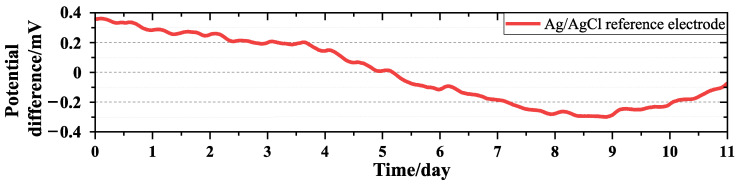
Time series of the potential difference of Ag/AgCl reference electrode.

During the testing of the potential stability of electrodes prepared with different double-pulse parameters, we also tested the potential difference of the Ag/AgCl reference electrode (218-type, Shanghai Leici) (Figure 8). We compared the potential stability of the electrode prepared using the double-pulse electrodeposition method with that of the Ag/AgCl reference electrode. It is evident that the potential stability of the reference electrode is inferior to that of the electrodes prepared by the double-pulse electrodeposition method with a positive direction peak current density of 10 $mA/{cm}^{2}$ and deposition times of 240 s, 260 s, 280 s, and 300 s.

Table 4 shows the potential stability parameters of other electrodes for marine electric field. It can be seen that the electrode which was prepared by double-pulse electrodeposition method with a positive direction peak current density of 10 $mA/{cm}^{2}$ and deposition time of 280 s has better potential stability.

### 3.4. The Noise Level of the Electrode

Electrochemical noise (EN) refers to the random non-equilibrium fluctuations of electrical state parameters (such as electrode potential, current density, etc.) that occur during the evolution of the electrochemical dynamic systems [38,39]. The Ag/AgCl electrode continuously undergoes electrochemical reactions in seawater, producing electrochemical noise. This is generated by the electrodes, rather than the test system or the external environment. To evaluate the noise level of the electrodes, it is necessary to determine the noise level of the test system, including both the instrument and environmental noise level. The noise level of the test system used in this article is 4.1 $nV/\sqrt{Hz}@1\;Hz$. The electrodes were connected to the test system, and their noise level along with the test system were measured. The noise level of the electrode can be expressed as [40]:

(5) $$Noise\;of\;the\;electrode=\sqrt{{noise\;of\;the\;test\;system\;and\;electrode}^{2}-{noise\;of\;the\;test\;system}^{2}}$$

**Figure 9:**
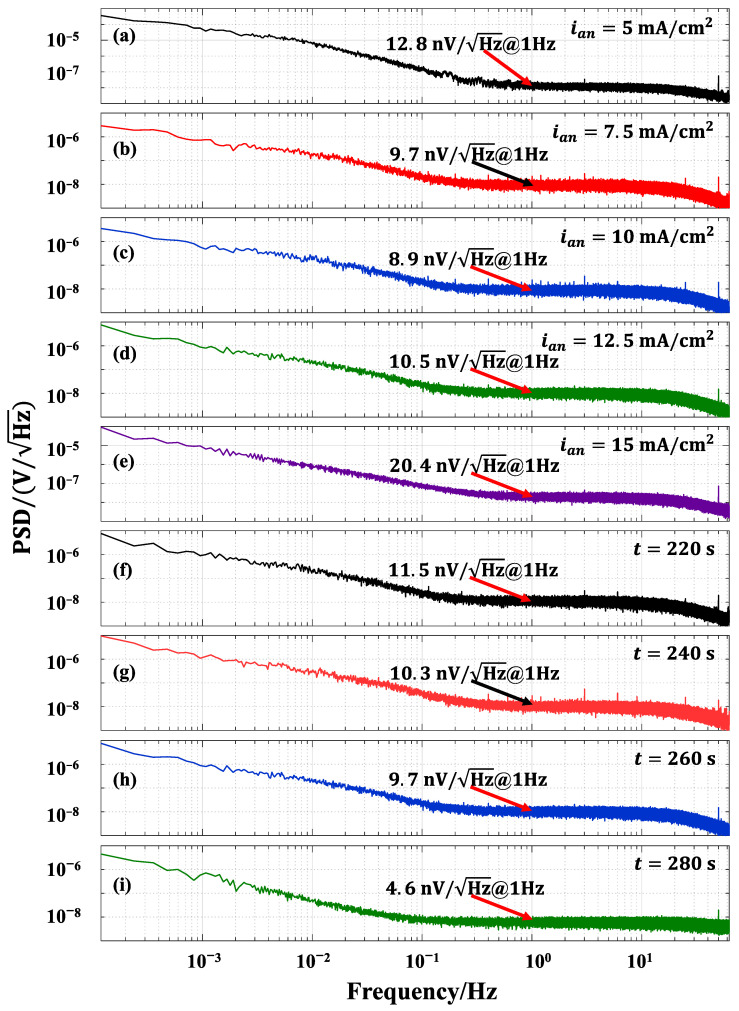
The power spectral density of test system and Ag/AgCl electrode plated at a deposition time of 300 s: (**a**) $i_{an}=5\;mA/{cm}^{2}$, (**b**) $i_{an}=7.5\;mA/{cm}^{2}$, (**c**) $i_{an}=10\;mA/{cm}^{2}$, (**d**) $i_{an}=12.5\;mA/{cm}^{2}$, (**e**) $i_{an}=15\;mA/{cm}^{2}$; noise density of Ag/AgCl electrode plated at a positive direction peak current density of $10\;mA/{cm}^{2}$: (**f**) t=220 s, (**g**) t=240 s, (**h**) t=260 s, (**i**) t=280 s.

For the test, the electrodes were immersed in 3.5% NaCl solution with 5 cm distance between the centers of the two electrodes. All electrode pairs were tested under the same experimental conditions, allowing for a comparison of noise levels between them. The noise level test results (the power spectral density, PSD) of the test system and all electrodes are shown in Figure 9, and the calculated electrode noise levels are shown in Table 5. It is evident from Table 5 that the noise level of the electrode varies depending on the double-pulse parameters used during preparation. The positive direction peak current density and deposition time are important factors affecting the electrode noise level. Among all electrodes tested, the Ag/AgCl electrode prepared with a deposition time of 280 s and a positive direction peak current density of 10 $mA/{cm}^{2}$ exhibited the lowest noise level. On the contrary, the Ag/AgCl electrode prepared at a deposition time of 300 s and a positive direction peak current density of 15 $mA/{cm}^{2}$ has the highest noise level. This is consistent with the results of the electrode’s stability test, which showed that electrodes with good potential stability also have relatively low noise levels.

Combined with the potential stability test results of the electrodes, it is evident that the noise level of the electrode is relatively high when the potential difference of the electrode pair is too large or when there is an abnormal bulge in the potential difference.

It seems that the potential difference and the potential difference drift of the electrode pair have an effect on the electrode’s noise level. From the previous analyses, it can be seen that the noise level of electrodes prepared under different conditions is also determined by the morphology of the electrode. Therefore, similar to the potential stability of the electrode pair, the electrode’s noise level is an important performance parameter that characterizes the performance of the electrode.

Table 6 displays the noise levels of different electrodes for marine electric fields. The test results indicated that the electrode prepared by double-pulse electrodeposition with a positive direction peak current density of 10 $mA/{cm}^{2}$ and a deposition time of 280 s exhibits a lower noise level. However, the noise level of this electrode is a bit higher compared to other electrodes. Therefore, the noise level of electrodes prepared by the double-pulse electrodeposition method needs improvement.

## 4. Conclusions

The performance of the Ag/AgCl electrode determines the quality of data collected in marine electric field measurements. However, the marine electric field test environment is complex and the marine electric field signal is weak. To achieve reliable marine electric field measurements, the basic performance of Ag/AgCl electrodes needs to be improved and controlled. There are many parameters that can be adjusted during the preparation of electrodes by double-pulse electrodeposition, which makes it very difficult to control the performance of the electrode. In this paper, we experimentally verified the effects of positive direction peak current density and deposition time on the electrode micromorphology, exchange current density, potential stability, and noise level to gain a deeper understanding of the influence of the double-pulse electrodeposition method on the performance of prepared electrodes.

In this article, the morphology and exchange current density of electrodes prepared with different double-pulse electrodeposition parameters were tested to evaluate the specific surface area and porosity of the electrode. We investigated the performance characteristics of electrodes prepared with different pulse parameters by testing the potential stability and noise level of the electrode, and the effect of the deposition layer’s morphology on electrode performance was studied. The test results indicate that the positive direction peak current density and deposition time are critical factors that affect electrode performance. Increasing the specific surface area and porosity of the deposition layer can lead to a larger exchange current density, better potential stability, and lower noise level. The electrodes prepared with a positive direction peak current density of 10 $mA/{\mathrm{cm}}^{2}$ and a deposition time of 280 s exhibit superior performance, and they had a smooth surface and a high exchange current density, a good potential stability, and low noise level.

The performance of electrodes prepared by the double-pulse electrodeposition method for marine electric field measurements in different sea areas needs further study. This may be a way to understand the impact of different electrode preparation schemes on the application performance of the resulting electrodes. This may not only have a positive impact on marine electric field measurements, but may also affect the use of this type of electrode in marine electric field measurement.

## Figures and Tables

**Table 1:** The double-pulse electrodeposition parameters for preparation of the electrode.

Positive Direction Parameters			Reverse Direction Parameters			Deposition Time
$i_{an}\;$($mA/{cm}^{2}$)	$t_{on}\;$(ms)	$t_{off}\;$(ms)	$i_{p}\;$($mA/{cm}^{2}$)	$t_{on}\;$(ms)	$t_{off}\;$(ms)	t (s)
5	12	4	5	3	1	300
7.5	12	4	5	3	1	300
10	12	4	5	3	1	300
12.5	12	4	5	3	1	300
15	12	4	5	3	1	300
10	12	4	5	3	1	280
10	12	4	5	3	1	260
10	12	4	5	3	1	240
10	12	4	5	3	1	220

**Table 2:** The characteristics of the particles in electrode’s deposition layers.

Electrode Number	Electrodeposition Parameters		The Sizes of Particles in Electrode’s Deposition Layers
	Positive Direction Peak Current Density *i_an_* (mA/cm^2^)	Deposition Time *t*(s)	
1 (Figure 3a)	5	300	Uniform in size and essentially less than 1.3 μm, but the deposited layer does not completely cover the silver foil.
2 (Figure 3b)	7.5	300	The size is basically less than 1 micron, but the shape varies greatly.
3 (Figure 3c)	10	300	The size is relatively uniform and basically smaller than 4.31 μm.
4 (Figure 3d)	12.5	300	Particle size is uneven, with more large-sized particles.
5 (Figure 3e)	15	300	The particle size is very uneven, with some particles reaching a size of 10 μm.
6 (Figure 3f)	10	220	Porous and small particle size, but agglomeration occurs.
7 (Figure 3g)	10	240	Particle size varies greatly.
8 (Figure 3h)	10	260	There is some porosity, but the size is uneven.
9 (Figure 3i)	10	280	There is some porosity, and the particle size is uniform and basically not larger than 1.42 μm.

**Table 3:** The potential stability parameters of the Ag/AgCl electrode.

Electrode Number	Electrodeposition Parameters		Potential Stability Parameters			
	Positive Direction Peak Current Density *i_an_* (mA/cm^2^)	Deposition Time *t*(s)	Potential Difference (mV)		Potential Difference Drift (mV/24 h)	
			Maximum	Minimum	Maximum	Minimum
1 (Figure 7a)	5	300	1.42	1.05	0.12	0.028
2 (Figure 7b)	7.5	300	−0.53	−0.78	0.071	0.010
3 (Figure 7c)	10	300	0.16	0.093	0.027	0.0059
4 (Figure 7d)	12.5	300	−0.92	−1.27	0.24	0.021
5 (Figure 7e)	15	300	8.76	8.0	0.27	0.051
6 (Figure 7f)	10	220	1.19	0.78	0.074	0.0074
7 (Figure 7g)	10	240	−0.20	−0.30	0.045	0.0052
8 (Figure 7h)	10	260	−0.30	−0.44	0.095	0.0062
9 (Figure 7i)	10	280	−0.0037	−0.079	0.027	0.0048

**Table 4:** The potential stability parameters of the electrode for marine electric field.

Electrode	Potential Difference		Minimum Value of Potential Difference Drift
	Maximum	Minimum	
Ag/AgCl electrode prepared by direct current electrolysis [6]	0.1 mV	Unknown	0.005 mV/24 h
Ag/AgCl prepared by sintering [9]	0.035 mV	Unknown	0.01 mV/24 h
Ag/AgCl prepared by electrospray [36]	0.056 mV	−0.051 mV	0.005 mV/100h
Ag/AgCl electrode pair on the template of carbon foam [5]	Unknown	Unknown	0.02 mV/24 h
Ag-AgNWs-CF electrode [8]	>0.1 mV	Unknown	0.06722 mV/24 h
MCNT/Ag/AgCl electrode [10]	0.1 mV	0.0363 mV	Unknown
3D Flower-Like Ag-CF electrode [14]	Unknown	0.03308 mV	0.01862 mV/24 h
rGO-CF electrode [37]	Unknown	0.04989 mV	0.05587 mV/24 h

**Table 5:** The noise level of the Ag/AgCl electrode.

Electrode Number	Electrodeposition Parameters		Noise Level	
	Positive Direction Peak Current Density *i_an_* (mA/cm^2^)	Deposition Time *t*(s)	$\mathrm{Test}\;\mathrm{System}\;\mathrm{and}\;\mathrm{Electrode}\;(nV/\sqrt{Hz}@1Hz$)	$\mathrm{Electrode}\;(nV/\sqrt{Hz}@1Hz$)
1 (Figure 9a)	5	300	12.8	12.1
2 (Figure 9b)	7.5	300	9.7	8.8
3 (Figure 9c)	10	300	8.9	7.9
4 (Figure 9d)	12.5	300	10.5	9.7
5 (Figure 9e)	15	300	20.4	20.0
6 (Figure 9f)	10	220	11.5	10.7
7 (Figure 9g)	10	240	10.3	9.4
8 (Figure 9h)	10	260	9.7	8.8
9 (Figure 9i)	10	280	4.6	2.1

**Table 6:** The noise level of the electrode for marine electric field.

Electrode	$\mathrm{Noise}\;\mathrm{Level}\;(nV/\sqrt{Hz}@1Hz$)
Ag/AgCl electrode prepared by direct current electrolysis [6]	0.6
Ag/AgCl prepared by sintering [9]	1.57
Ag/AgCl prepared by electrospray [36]	2.48
Ag/AgCl electrode pair on the template of carbon foam [5]	1.6
Ag-AgNWs-CF electrode [8]	1.03
MCNT/Ag/AgCl electrode [10]	4.1
3D Flower-Like Ag-CF electrode [14]	0.92
rGO-CF electrode [37]	0.83
Multi-rod Type Ag–AgCl [41]	3.7

## Data Availability

The data presented in this study are available on request from the corresponding author.
